# Effect of Zearalenone and Hormone Regulators on Microspore Embryogenesis in Anther Culture of Wheat

**DOI:** 10.3390/plants8110487

**Published:** 2019-11-10

**Authors:** Dorota Weigt, Janetta Niemann, Idzi Siatkowski, Joanna Zyprych-Walczak, Przemysław Olejnik, Danuta Kurasiak-Popowska

**Affiliations:** 1 Department of Genetics and Plant Breeding, Poznań University of Life Sciences, 11 Dojazd St., 60–632 Poznań, Poland; 2 Department of Mathematical and Statistical Methods, Poznań University of Life Sciences, 28 Wojska Polskiego St., 60–637 Poznań, Poland

**Keywords:** zearalenone, microspore embryogenesis, androgenic structures, doubled haploid, wheat

## Abstract

The purpose of this work was to assess the impact of zearalenone (ZEN) and selected hormone regulators on the effectiveness of microspore embryogenesis in anther culture of wheat. The plant material comprised F_1_ hybrids of winter and spring wheat. Six combinations of media inducing microspore proliferation and formation of embryogenic structures were investigated: two combinations of growth regulators (D - 2,4-D + dicamba, K - 2,4-D + kinetin), each with three ZEN concentrations (0 mL/L, 0.1 mL/L, 0.2 mL/L). A significant increase in microspore embryogenesis effectiveness on media with the addition of ZEN was observed both at the stages of its induction and the formation of green plants in some genotypes. In case of both combinations of growth regulators, an increased concentration of ZEN resulted in more effective induction of microspore embryogenesis. The most effective induction medium was the D medium supplemented with 0.2 mL/L ZEN. As a result of the use of zearalenone together with two combinations of growth regulators, all genotypes tested produced androgenic structures, which indicates the breakdown of genotypic recalcitrant in the analysed hybrids. In addition, green plants were obtained from 18 out of 19 tested hybrids. The addition of ZEN to the medium did not affect the number of regenerated albino plants nor the number of spontaneous genome doublings proportion.

## 1. Introduction

Microspore embryogenesis is a process in which the embryological development of microspores leads to regeneration of haploid plants [1]. The technological value of these plants is significant as they arise from cells following meiotic division and thus have unique gene combinations [2]. As a result of spontaneous or induced doubling of the haploid genome, fertile and fully homozygous plants are produced. The greatest asset of this method is its potential; one anther holds over a thousand microspores and in theory each of them can give rise to a new plant [3] while obtaining haploid plants from a single flower in all other methods (e.g., cross breeding with corn) limits production to one plant per floret [4]. Haploids are widely used in genetic research, such as mapping quantitative features (QTL), gene mapping, associative mapping [5], genetic transformation [6], as well as genetic engineering [7]. Moreover, doubled haploid (DH) technology is a valuable tool in plant breeding, including wheat [8].

Effectiveness of microspore embryogenesis (the ratio of obtained plants to the number of placed anthers) is dependent on multiple factors, of which the following are considered key: the donor plants’ genotype, microspores’ development stage when starting the culture, preliminary treatment of microspores in attempt to switch their development pathway from the gametophytic to sporophytic, and composition of the induction medium—especially the content of growth regulators [9,10,11]. Genotype dependency and albinism are the most important limiting factors in the method of obtaining DH based on microspore embryogenesis [11]. For that reason, it is crucial to identify a factor which would improve the effectiveness of wheat anther culture in breaking the recalcitrance or limiting yield of albino plants. 

Zearalenone (ZEN), 6-(10-hydroxy-6-oxo-trans–1–undecenyl)-b-resorcylic acid lactone, was first isolated from extracts of fungus Gibberella zeae (*Fusarium graminearum*) by Stob et al. (1962) [12]. ZEN is a mycotoxin produced mainly by common soil fungi belonging to the genus Fusarium, which are common contaminants of cereal crops worldwide [13]. This compound (previously known as F-2 toxin) is a nonsteroidal estrogenic metabolite biosynthesized through a polyketide pathway and is an endogenous regulator of the sexual stage of development of fungi [14]. Its occurrence in feed is frequently implicated in reproductive disorders of farm animals and occasionally in hyperoestrogenic syndromes in humans [15]. Moreover, some experiments show that ZEN can also act as a phytohormone and in low concentrations can influence the development and yield of crop plants [16]. It has been shown that exogenous application of ZEN and its derivatives can stimulate generative development in plants, which suggests its participation in the mechanism of flowering. Watering and soaking wheat and soybean grains with ZEN solution resulted in higher yields of these plants. Meng et al. (1992) [17] suggested that ZEN has a stimulating effect on flowering induction and can partly replace the low temperature requirement for vernalisation in winter wheat. An increase in endogenous ZEN during vernalisation was also recorded by Fu and Meng (1994) [18] in many winter plants.

The effect of ZEN was similar to the activity of auxins in in vitro cultures. Treatment with ZEN had an impact on calli proliferation and cell differentiation. For example, ZEN stimulated the initiation of the vegetative bud in tobacco pith callus tissue [19]. Biesaga-Kościelniak et al. (2003) [20] stated that ZEN could be used in the maize pollination system of haploid production to ovary treatment. Used in an appropriate concentration, it stimulates indirect regeneration and increases the frequency of embryo formation. Therefore, in the present study we examine the influence of ZEN on induction of microspore embryogenesis (androgenic structures formation), green and albino plant regeneration, as well as spontaneous doubled haploid formation in anther culture of wheat, where indirect regeneration is observed and a high yield of embryos is desirable.

## 2. Results

### 2.1. Influence of ZEN on Androgenic Structures Formation 

**Figure 1:**
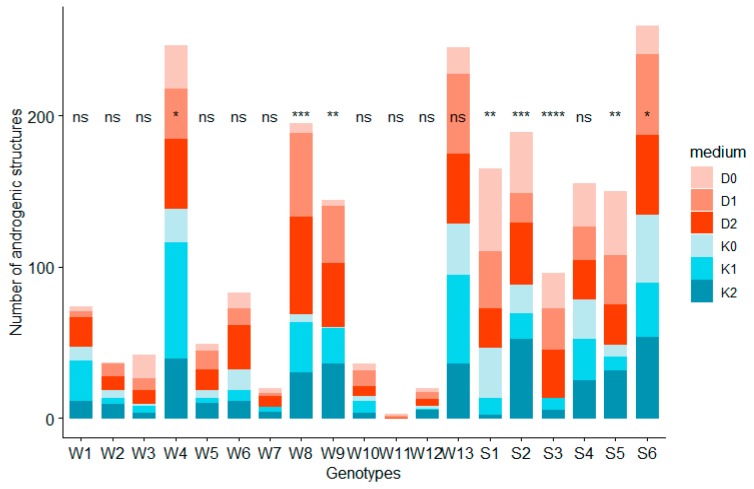
The effect of the genotype on the number of androgenic structures obtained. Significance code: ‘****’ 0.0001; ‘***’ 0.001; ‘**’ 0.01; ‘*’ 0.05 and ns—not significant indicates statistical differences in the number of AS received on different media (D0, D1, D2, K0, K1, K2) within one genotype. Genotypes: W1–W13 were winter phenotypes, S1–S6 were spring phenotypes.

Androgenic structures were observed on anthers of all analysed hybrids (Figure 1). W4, W8, W13, S2, S6 were the five genotypes that produced the most AS, where the efficiency of microspore embryogenesis induction was above 10androgenic structures per 100 placed anthers. Microspores of those genotypes showed a positive reaction when ZEN was added to the induction medium, increasing the number of AS (Table 1). When ZEN was applied, anthers of hybrids W8 and W12 produced AS over ten times more effectively than anthers placed on medium without zearalenone. The EI of these genotypes was not dependent on other regulatory hormones present in the induction medium (Table 1). On the contrary, genotype S1 showed a decrease in effectiveness of microspore embryogenesis induction with the increase of ZEN concentration, in relation to the induction level on D0 and K0 media. Such different responses of hybrids to the composition of the media and growth regulators as well as ZEN are a result of a strong influence of the genotype on microspore embryogenesis induction. 

**Figure 2:**
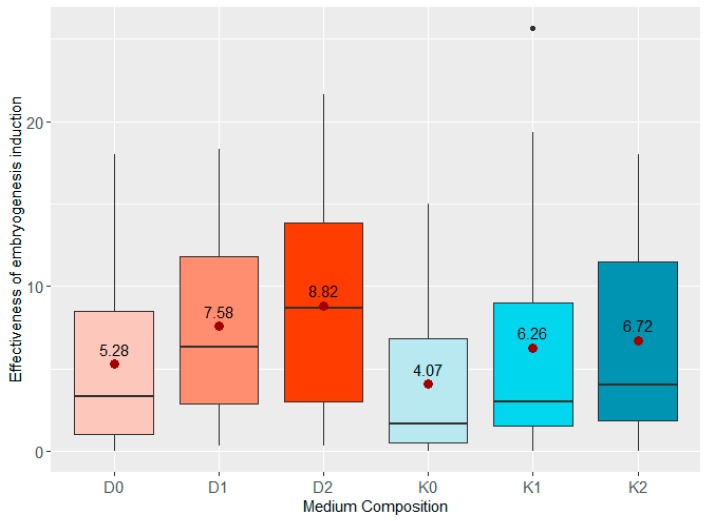
Boxplots of the effect of growth regulators and various concentrations of ZEN on the effectiveness of microspore embryogenesis induction of all analyzed genotypes together. “
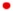
“—the average effectiveness expressed as the number of androgenic structures per 100 placed anthers.

Medium D2 containing 2,4-D, dicamba and the highest ZEN concentration (0.2 mL/L) turned out to be the most effective medium, where the effectiveness of regeneration reached 8.82 AS per 100 placed anthers (on average for all analysed genotypes) (Figure 2). Medium D as well as medium K induced microspore embryogenesis most effectively when the highest concentration of ZEN (0.2 mL/L) was used.

**Figure 3:**
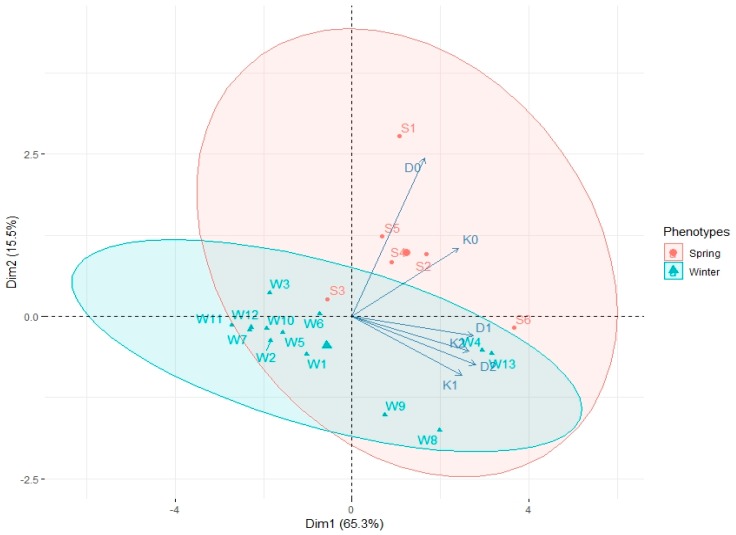
PCA biplot analysis showing the influence of growth regulators and various concentrations of ZEN in the media composition (vectors: D0, D1, D2, K0, K1, K2) on microspore embryogenesis induction of spring (S1–S6) and winter (W1–W13) wheat hybrids. Ellipsoids indicated 80.8% confidence.

The PCA analysis showed that most spring genotypes (S1, S2, S4, S5) were not stimulated by ZEN, because stronger correlation between AS formation by these hybrids and media D0 and K0 occurred (Figure 3). On the contrary, most winter genotypes were observed around the vectors representing ZEN-enhanced media, which means that ZEN has a bigger influence on EI in case of winter genotypes. Moreover, it can be observed that media containing ZEN had the greatest influence on EI of W4, W8, W9 and W13 winter genotypes, as the points representing these genotypes found on Figure 3 oscillate around D1, D2, K1 and K2 vectors.

### 2.2. Influence of ZEN on Green Plant Regeneration 

**Figure 4:**
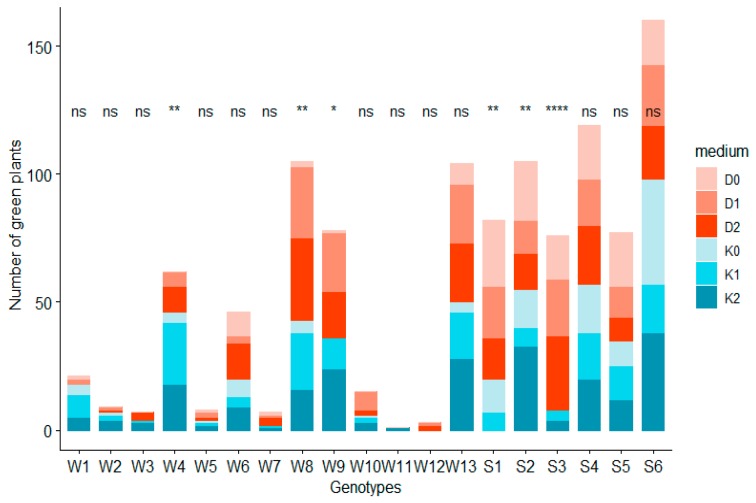
The effect of the genotype on the number of green plants obtained. Significance code: ‘****’ 0.0001; ‘***’ 0.001; ‘**’ 0.01; ‘*’ 0.05 and ns—not significant indicates statistical differences in the number of green plants received on different media within one genotype. Genotypes: W1–W13 were winter phenotypes, S1–S6 were spring phenotypes.

Similar relationships were observed in green plant production and AS. Reactions of specific genotypes to growth regulators and ZEN varied. Within winter genotypes most green plants were regenerated from AS of W8 and W13 genotypes, for which the GPR effectiveness equalled to 5.8 plants per 100 placed anthers (Figure 4). Simultaneously, a rise in GP regeneration in the earlier stated genotypes, related to an increase of ZEN on the induction medium, was observed (Table 2). Spring genotypes, which on average produced more GP than winter genotypes, regenerated most effectively from anthers of S6 hybrid (GPR was equal to 8.9 green plants per 100 placed anthers). It must be stated, though, that the influence of ZEN on S6 was significantly weaker than on W8 or W12 genotypes. This tendency was characteristic for most spring and winter genotypes, which is shown in Table 2. 

Moreover, it was observed that W3 and W12 hybrids with low GPR underwent microspore embryogenesis induction only when ZEN was present in the medium: no plants were produced on the D0 and K0 media. This result suggests that the recalcitrance of those genotypes can be overcome as a result of ZEN acting on their anthers. This relationship has been observed in other genotypes but only in one of the applied combinations of hormone content in the medium (Table 2).

**Figure 5:**
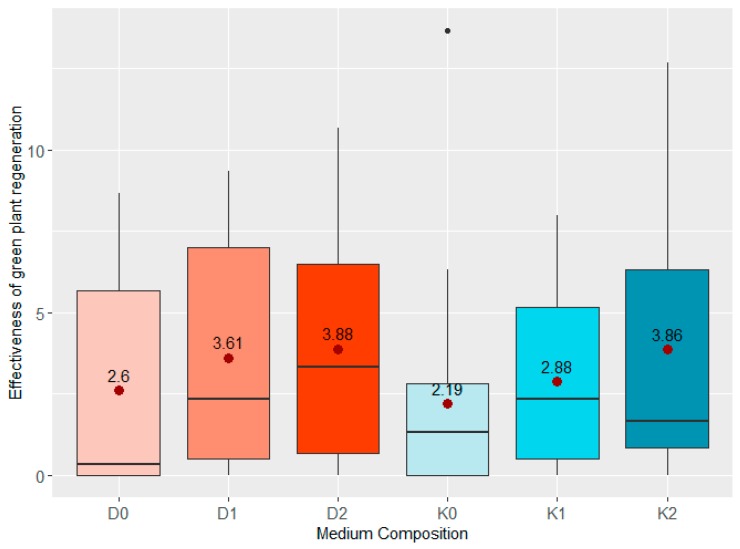
Boxplots of the effect of growth regulators and various concentrations of ZEN on the effectiveness of green plant regeneration of all analyzed genotypes together. “
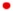
“—the average effectiveness expressed by the number of green plants per 100 placed anthers.

**Figure 6:**
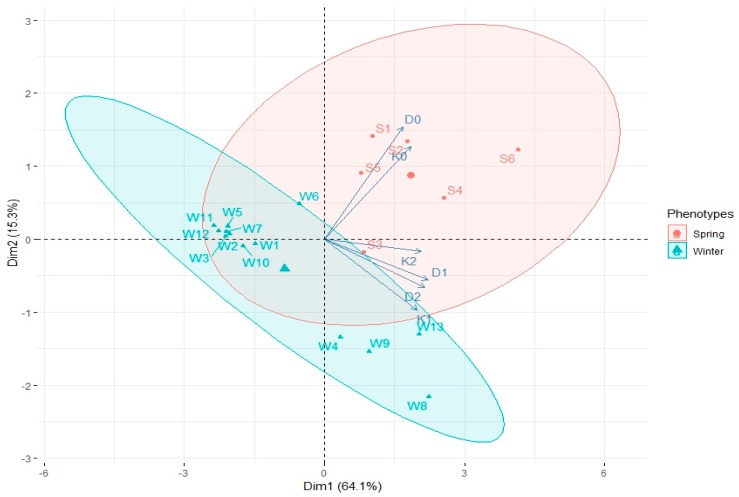
PCA biplot showing the influence of growth regulators and various concentrations of ZEN in the media composition (vectors: D0, D1, D2, K0, K1, K2) on green plant regeneration of spring (S1–S6) and winter (W1–W13) wheat hybrids. Ellipsoids indicated 79.4% confidence.

Average production of green plants for all analysed genotypes was highest in media supplemented with D2 and K2 containing 0.2 mL/L ZEN. Interestingly, the GPR values were similar in those media (3.88 and 3.86, respectively), whereas induction of embryogenesis that predated green plant production was much more effective on D2 rather than on K2 (Figure 5). PCA made it possible to differentiate two main phenotypic groups—spring and winter hybrids. The reactions of these groups to ZEN present in the induction media were not alike. Winter hybrids: W4, W8, W9, W13 (points oscillating around the vectors marked with ZEN induced combinations D1, D2, K1, K2 on the graph) were characterised by a higher GPR on media supplemented with ZEN (Figure 6). On the contrary, spring hybrids were less stimulated by ZEN added to the medium. It can be seen on Figure 6 that most spring genotypes were observed around D0 and K0 vectors.

### 2.3. Influence of ZEN on Albino Plant Regeneration 

**Figure 7:**
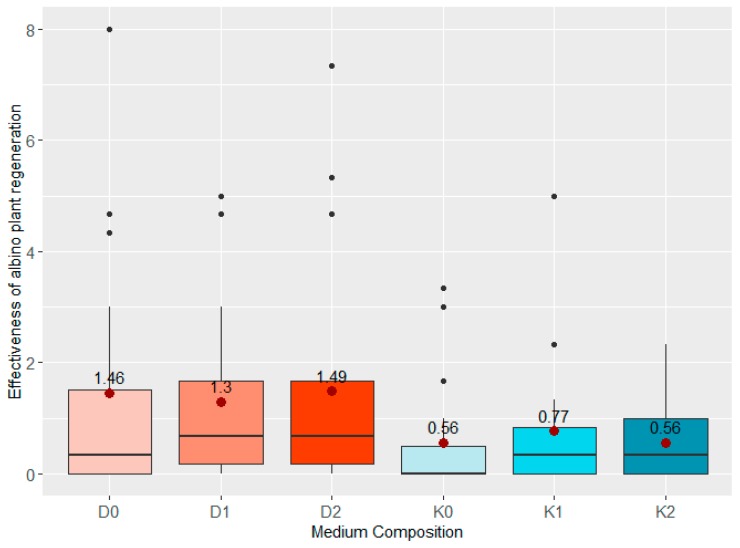
Boxplots of the effect of growth regulators and various concentrations of ZEN on the effectiveness of albino plant regeneration of all analyzed genotypes together. “
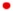
“—the average effectiveness expressed by the number of albino plants per 100 placed anthers.

**Figure 8:**
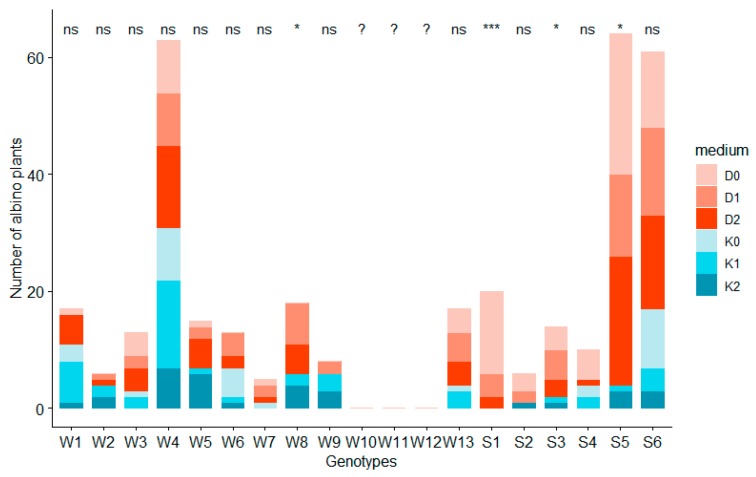
The effect of the genotype on the number of albino plants obtained. Significance code: ‘****’ 0.0001; ‘***’ 0.001; ‘**’ 0.01; ‘*’ 0.05 and ns—not significant indicates statistical differences in the number of albino plants received on different media within one genotype. Genotypes: W1–W13 were winter phenotypes; S1–S6 were spring phenotypes.

Among the investigated F_1_ hybrids, three (W10, W11, W12) did not produce any albino plants. Production of AP on anthers of other hybrids, however, did not depend on the presence of ZEN in the medium (Table 3). The mean APR value was higher in case of the medium supplemented with 2,4-D and dicamba (D) than in case of the medium supplemented with of 2-4-D and kinetin (K) (Figure 7). Analysis of albino plants regeneration and PCA proved that APR is influenced by the genotype of donor plant mainly (Table 3, Figure 8) as well as the addition of growth regulators (Figure 7) but by the latter to a smaller extent.

**Figure 9:**
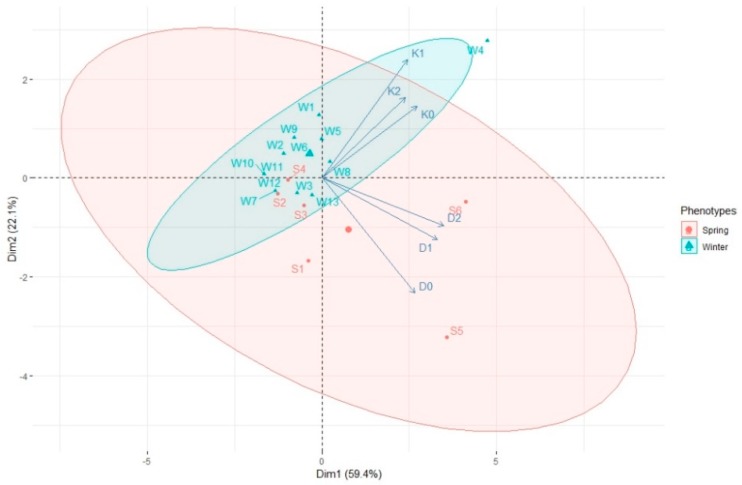
PCA biplot showing the influence of growth regulators and various concentrations of ZEN in the media composition (vectors: D0. D1. D2. K0. K1. K2) on albino plant regeneration of spring (S1–S6) and winter (W1–W13) wheat hybrids. Ellipsoids indicated 81.5% confidence.

PCA shows different grouping of vectors and genotypes than EI and GPR—the vectors representing kinetin supplemented medium (K0, K1, K2) are correlated to each other and mainly winter genotypes are found around them, unlike dicamba supplemented medium vectors (D0, D1, D2), around which spring genotypes are found (Figure 9). The correlation was not, however, very strong (most genotypes are found on the opposite side of the axis on the graph, which could indicate a stronger influence of APR genotype than of biologically active substances found in the induction medium).

### 2.4. Influence of ZEN on Spontaneous Doubled Haploid Formation 

**Figure 10:**
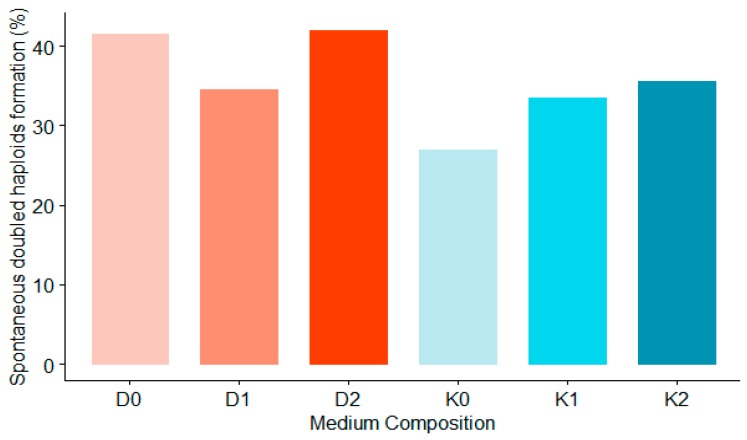
The effect of growth regulators and various concentrations of ZEN on the spontaneous doubled haploids formation expressed as percentages of the whole population of regenerated green plants.

ZEN did not have a significant influence on ploidy level (PL) in the obtained plants. The number of plants that spontaneously doubled their chromosome numbers was rather dependant on the general number of obtained plants. Spontaneous doublings were however more frequent observed on the D media, independent of ZEN concentration. The highest DHF was observed on D0 and D2 media (over 40%), as shown on Figure 10. 

## 3. Discussion

Numerous studies have shown that one of the most important steps in microspore embryogenesis is induction; it means initiation of autonomous embryo development in microspore cells in response to external and/or internal signals. Immature microspores under microspore embryogenesis require extensive genetic reprogramming before embriogenesis can begin because their embryogenic program is under strong epigenetic repression [21]. Stress and hormones influence chromatin reorganisation, which is a consequence of reprogramming transcription, and translation profiles in cells [22] play a key role in microspore embryogenesis induction [23]. Activation of the reprogramming process should result in initiation of embryogenic program and not development of non-morphogenic calli or apoptosis of microspores [24]. Moreover, in microspore embryogenesis, stress treatment initiates a rearrangement of the cytoskeleton and re-localisation of the nucleus. The nucleus moves to the centre of the cell where it is surrounded by cytoplasmic strands. This way star-like structures, which are considered a first sign of embryogenic induction, are formed. In this experiment, microspores of all 19 analysed hybrids underwent induction, showing effective cell stimulation and genetic recalcitrance overcoming as a result of the use of different variations of induction medium. Moreover, the development of androgenic structures and later plants was observed in 18 out of 19 analysed genotypes.

Auxinic herbicide 2,4-D (2,4-dichlorophenoxyacetic acid) is the most widely applied microspore embryogenesis inducer [24,25]. According to literature, this synthetic hormone when added to an induction medium has auxin-like effects and in addition acts as a stress factor [26]. Its concentration in the induction medium should be in the range of 0.5–2.0 mg/L. Too high a concentration may result in loss of the calluses’ ability to regenerate into a plant as the stress hormones accumulate and suppress further development [27]. On the contrary, a concentration which is too low does not induce the embryogenic response at all [28]. Seldmirova et al. (2016) [16] reported that auxin gradients are important in setting up embryo symmetry and that the right ratio of endo and exogenous auxins in the microspores determined the differentiation of the embryoids.

The type and length of exposure to a stress factor affect the accumulation of endogenic auxins (mainly IAA). Knowing this, it is important to properly adjust the conditions of primary treatment and the levels of hormones added to the induction medium such that the total concentration of all auxins within a cell is optimised. In research regarding wheat microspore embryogenesis, growth regulators other than 2,4-D were also added to the medium such as: dicamba, picloram, kinetin [29,30], BAP [30,31] and PAA [32,33].

The ratio of added growth and plant development hormones also affect the in vitro culture cells in artificial conditions. In this experiment the medium contained 2,4-D and was additionally supplemented with auxin in a form of dicamba or cytokine in a form of kinetin. These hormones were chosen for the experiment based on literature and personal research [34,35,36]. Due to a strong influence of genotype on EI and GPR, it may seem that using these two solutions in one experiment could have a positive influence on the average effectiveness of microspore embryogenesis. Most genotypes were greater induced to proliferation and embryogenesis when under the influence of a medium containing only auxin; however, some responded noticeably better when kinetin was also present in the medium. 

In the experiment 6 out of 19 analysed F_1_ hybrids (W1, W2, W4, W13, S4, S6) gave a higher average yield of AS, and 6 genotypes (W1, W2, W3, W4, S2, S6) regenerated more GP on kinetin supplemented medium than on the 2-4-D and dicamba supplemented medium. Earlier research carried out by the team also showed similar results when microspores of some genotypes proved to be embryogenic only on one out of two media, meanwhile being fully recalcitrant on the second (non-published data). It was moreover observed that EI happened more effectively on a D medium, the GPR level however was comparable on both media, which clearly shows the positive effect of kinetin on embryoid formation and further yield of plants. It was also noted that kinetin positively decreased the number of plants with chlorophyll defects, whose presence was 2 to 3 times more scarce on the kinetin supplemented medium in comparison to D medium. 

Apart from widely used growth regulators in the induction medium ZEN was used as well. Zearalenone just like 2.4-D is a stress factor and has auxin-like effects [37]. The stress is a result of cytotoxicity of ZEN and its non-steroid character that allows for modification of the structure of cell membranes by fusion of this compound into the lipid layer [38]. Gzyl-Malcher et al. (2017) [38] showed that calli cultured on ZEN containing medium were characterised by higher fatty acid saturation of lipid fractions than those grown in control medium. This is a proof of ZEN’s influence on the presence of oxidative stress in cells treated with this compound. 

During the induction of microspore embryogenesis, stress is thought to be a crucial factor for reprogramming gametophytic pathway in change of microspores into sporophytic types. ZEN in the study was the main factor responsible for the increase in EI of the winter hybrids, which could be a result of different reactions of winter and spring genotypes to the stress caused by low temperature. It is possible that primary treatment of anthers with a cold temperature might not be sufficient enough for effective induction of microspore embryogenesis of winter plants, which are characterised by higher tolerance of low temperatures unlike spring plants. Moreover, introduction of addiction factor to the medium, for instance, in the form of ZEN, leads to more effective reprogramming the development pathway of winter genotypes microspores and as a result more efficient formation of embriogenic structures. ZEN also impacts plant cells by stimulating their division and all processes related to differentiation, especially during embriogenesis pathway of indirect regeneration [14,20,39]. These observations are proved by our experiment in which ZEN had been used in anther cultures of wheat and turned out to be beneficial. The treatment with ZEN resulted in a significant increase of the effectiveness of induction of microspore embryogenesis for the investigated wheat hybrids, noting that supplementation with the concentration of 0.2 mL/L on average per all hybrids altogether stimulated microspores to divide more efficiently.

The difference in effectiveness of microspore embryogenesis induction under the influence of ZEN was visible mainly on all media supplemented with dicamba, where a positive correlation was observed (average value of EI for all tested hybrids: D0—0.53; D1—0.76; D2—0.88 rised with the increase of concentration of dicamba in the medium). This result proves that when picking the composition of the medium, one needs to take into consideration their ratios. Moreover, some plant hormones or biologically active substances may have a stronger impact on plant when cross-reacting.

Analysing the reaction of specific genotypes to ZEN, an increase of effectiveness of microspore embryogenesis induction can be seen when the compound is added to the medium. For some genotypes this increase was much higher than the effectiveness of microspores induction without ZEN stimulation. For the W8 genotype, over ten times increase was observed in the effectiveness of induction on the medium with the addition of 2.4-D and dicamba (from 2.0 to 21.67 respectively on D0 and D2 medium). W9 hybrid microspores underwent regeneration 37 times more effectively on K2 medium in comparison to K0, where the EI was equal to 12.33 and 0.33, respectively. 

## 4. Materials and Methods

### 4.1. Plant Material and Growth Condition

Donor material which has been used for anther cultures was genetically diversified. It was made up of 13 F_1_ winter wheat hybrids: W1(Thatcher x Chinese),W2 (GSTR 420 x Choptank), W3 (Wichita x KS96WGRC36), W4 (TAM 107 x Clark), W5 (Karl x Choptank), W6 (Clark x Choptank), W7 (KS96WGRC36 x NC8889 – 2A), W8 (Century x Wichita), W9 (Geneva x Century), W10 (Augusta x TAM 107), W11 (Freedom x Ok 101), W12 (Antelope x Lr 1), W13 (Clark x Klasic) and 6 F1 spring wheat hybrids: S1 (T-68x DC), S2 (T71x DC356/08-4-5/09), S3 (Arabella x T71), S4 (Tybalt x T71), S5 (Mandaryna x T71), S6 (Toridon x T71), that were earlier obtained in Department of Genetics and Plant Breeding Poznań University of Life Sciences. Plants were sown on an experimental field: the winter variety in mid-October of the previous year and the spring variety in mid-April of the same year that the anther cultures were placed.

### 4.2. In Vitro Culture Conditions

Spikes were collected when most of the microspores found inside anthers achieved medium or late uninuclear stage. The development stage of microspores was identified using a smear with 0.5% acetocarmine staining under a light microscope. Microspore embryogenesis was initiated by pre-treatment of the spikes at 4 °C. After one week of thermal stress, the spikes were sterilized for 4 min with 4.85% calcium hypochlorite (NaClO) for 4 min. They were then rinsed three times with sterile water for 5 min. Under sterile conditions, 1800 anthers were isolated from each genotype and placed on Petri dishes containing C17 [40] induction medium with slight modifications. Each Petri dish contained exactly 50 anthers that had been isolated from a single ear. Composition of the C17 medium was modified by adding 90 g/L maltose instead of 30 g/L sucrose, solidified using gerlite, and enriched with growth hormones. Two combinations of growth hormones for the induction medium were used: 2,4-D + dicamba and 2,4-D + kinetin, each of which had three concentrations (0 mL/L; 0.1 mL/L; 0.2 mL/L) of ZEN (Sigma-Aldrich®, Z2125). All treatments with growth regulators are presented in Table 4.

Petri dishes with anthers were incubated in darkness at 28 °C for 6–8 weeks. Androgenic structures (AS) which appeared after that period were transferred to MS [41] regeneration medium enhanced with 0.5 mg/L 1-naphtaleneacetic (NAA) and 0.5 mg/L kinetin.

Regeneration took place under artificial light (16-hour light/8-hour darkness) in the culture chamber at 24 °C. After 2–4 weeks plantlets were transferred to glass flasks containing MS medium with no additional growth regulators. The ploidy level of regenerated green plants was analysed by laser flow cytometry according to the method described by Śliwińska (2008) [42].

### 4.3. Data Analysis

The experiments were analysed as completely randomized designs. There were six repetitions of each treatment. A single repetition consisted of one Petri dish with 50 anthers originating from one spike. The results were analysed at three stages of the experiment: 8 weeks after anthers were placed on the induction medium, quantifying AS appearing on the anthers that represents microspore embryogenesis induction; 4 weeks after transfer AS to regeneration medium, quantifying green (GP) and albino (AP) plants; and during the transfer of GP to soil by analysing their ploidy level (PL). Then, the obtained quantitative data was used to calculate the effectiveness of microspore embryogenesis induction (EI = AS per 100 placed anthers), regeneration effectiveness of GP and AP (GPR = GP per 100 placed anthers and APR = AP per 100 placed anthers, respectively), and doubled haploid formation (DHF—spontaneous doubling of haploid genome as a percentage of the whole population of regenerated GP).

In the first stage of analysis, analysis of variance (ANOVA) for a two-factor linear model with interaction and Tukey’s HSD multiple comparison procedure were used. Principal Component Analysis (PCA) and a PCA biplot [43] were used to assess the influence of ZEN and growth hormone combinations in the induction medium on the microspore embryogenesis of winter and spring hybrids of wheat. Statistical calculations were performed with R software, version 3.5.2 (R Core Team 2018) [44].

## 5. Conclusions

Zearalenone had not been previously used in wheat anther cultures; it had only been used as a substance decreasing vernalization time in different varieties of winter plants. Due to its similarity to auxin (when it comes to the activity) as well as stress-inducing properties, ZEN can affects even a single nuclei microspore during microspore embryogenesis. The influence was analysed together with two different combinations of growth regulators in the induction medium and allowed to derive a conclusion that ZEN has stimulating properties on anther cultures. In the medium supplemented with ZEN, a more efficient microspore embryogenesis induction was observed as well as an increase of SA and GP numbers and an increase in microspore embryogenesis effectiveness as a result. This tendency was related to both concentrations of ZEN: 0.1 mL/L and 0.2 mL/L although the higher concentration of zearalenone was more effective. D2 medium with the addition of 2.4-D dicamba and 0.2 mL/L ZEN was the most optimal variant analysed in the experiment. Application of two variants of growth regulators in the induction medium and the addition of ZEN allowed to overcome the genotype recalcitrance inducting microspore embryogenesis from anthers of all investigated hybrids. Simultaneously, no influence of ZEN on the number of albino plants or the number of random genome doubling were observed.

## Figures and Tables

**Table 1:** Effectiveness of microspore embryogenesis induction of studied hybrids depending on medium composition expressed as the number of androgenic structures per 100 placed anthers.

Genotype		No. of Placed Anthers	Treatment					
			D0	D1	D2	K0	K1	K2
W1	Century x Wichita	1800	1,00	1,33	6,33	3,00	9,00	4,00
W2	Augusta x Tam 107	1800	0,00	3,00	3,00	1,67	1,33	3,33
W3	Freedom x Ok 101	1800	5,00	2,67	3,00	0,33	1,67	1,33
W4	Antelope x Lr 1	1800	9,33	11,00	15,33	7,33	25,67	13,33
W5	Geneva x Century	1800	1,33	4,00	4,67	1,67	1,00	3,67
W6	Clark x Choptank	1800	3,33	3,67	9,67	4,67	2,33	4,00
W7	Klasic x Clark	1800	1,00	0,67	2,33	0,00	1,00	1,67
W8	Thatcher x Chińska	1800	2,00	18,33	21,67	1,67	11,00	10,33
W9	GSTR 420 x Choptank	1800	1,00	12,67	14,00	0,33	7,67	12,33
W10	Wichita x KS96WGRC36	1800	1,33	3,33	2,33	1,00	2,67	1,33
W11	Tam 107 x Clark	1800	0,33	0,33	0,33	0,00	0,00	0,00
W12	Karl 92 x Choptank	1800	0,67	1,67	1,33	0,67	0,33	2,00
W13	KS96WGRC36 x NC8889-2A	1800	5,67	17,67	15,33	11,33	19,33	12,33
S1	Arabella x T71	1800	18,00	12,67	8,67	11,00	3,67	1,00
S2	Tybalt x T71	1800	13,33	6,33	13,67	6,33	5,67	17,67
S3	Mandaryna x T71	1800	7,67	9,00	10,67	0,00	2,67	2,00
S4	Toridon x T71	1800	9,33	7,33	8,67	8,67	9,00	8,67
S5	T68xDC356/08-4-5/09	1800	14,00	10,67	9,00	2,67	3,00	10,67
S6	T71xDC356/08-4-5/09	1800	6,00	17,67	17,67	15,00	12,00	18,00

**Table 2:** Effectiveness of green plants regeneration of studied hybrids depending on medium composition expressed as the number of green plants per 100 placed anthers.

Genotype		No. of Placed Anthers	Treatment					
			D0	D1	D2	K0	K1	K2
W1	Century x Wichita	1800	0,33	0,67	0,00	1,33	3,00	1,67
W2	Augusta x Tam 107	1800	0,00	0,33	0,33	0,33	0,67	1,33
W3	Freedom x Ok 101	1800	0,00	0,00	1,00	0,00	0,33	1,00
W4	Antelope x Lr 1	1800	0,00	2,00	3,33	1,33	8,00	6,00
W5	Geneva x Century	1800	0,33	0,67	0,33	0,33	0,33	0,67
W6	Clark x Choptank	1800	3,00	1,00	4,67	2,33	1,33	3,00
W7	Klasic x Clark	1800	0,33	0,33	1,00	0,00	0,33	0,33
W8	Thatcher x Chińska	1800	0,67	9,33	10,67	1,67	7,33	5,33
W9	GSTR 420 x Choptank	1800	0,33	7,67	6,00	0,00	4,00	8,00
W10	Wichita x KS96WGRC36	1800	0,00	2,33	0,67	0,33	0,67	1,00
W11	Tam 107 x Clark	1800	0,00	0,00	0,00	0,00	0,00	0,00
W12	Karl 92 x Choptank	1800	0,00	0,33	0,67	0,00	0,00	0,00
W13	KS96WGRC36 x NC8889-2A	1800	2,67	7,67	7,67	1,33	6,00	9,33
S1	Arabella x T71	1800	8,67	6,67	5,33	4,33	2,33	0,00
S2	Tybalt x T71	1800	7,67	4,33	4,67	5,00	2,33	11,00
S3	Mandaryna x T71	1800	5,67	7,33	9,67	0,00	1,33	1,33
S4	Toridon x T71	1800	7,00	6,00	7,67	6,33	6,00	6,67
S5	T68xDC356/08-4-5/09	1800	7,00	4,00	3,00	3,33	4,33	4,00
S6	T71xDC356/08-4-5/09	1800	5,67	8,00	7,00	13,67	6,33	12,67

**Table 3:** Effectiveness of APR of studied hybrids depending on medium composition expressed as the number of AP per 100 placed anthers.

Genotype		No. of Placed Anthers	Treatment					
			D0	D1	D2	K0	K1	K2
W1	Century x Wichita	1800	0,33	0,00	1,67	1,00	2,33	0,33
W2	Augusta x Tam 107	1800	0,00	0,33	0,33	0,00	0,67	0,67
W3	Freedom x Ok 101	1800	1,33	0,67	1,33	0,33	0,67	0,00
W4	Antelope x Lr 1	1800	3,00	3,00	4,67	3,00	5,00	2,33
W5	Geneva x Century	1800	0,33	0,67	1,67	0,00	0,33	2,00
W6	Clark x Choptank	1800	0,00	1,33	0,67	1,67	0,33	0,33
W7	Klasic x Clark	1800	0,33	0,67	0,33	0,33	0,00	0,00
W8	Thatcher x Chińska	1800	0,00	2,33	1,67	0,00	0,67	1,33
W9	GSTR 420 x Choptank	1800	0,00	0,67	0,00	0,00	1,00	1,00
W10	Wichita x KS96WGRC36	1800	0,00	0,00	0,00	0,00	0,00	0,00
W11	Tam 107 x Clark	1800	0,00	0,00	0,00	0,00	0,00	0,00
W12	Karl 92 x Choptank	1800	0,00	0,00	0,00	0,00	0,00	0,00
W13	KS96WGRC36 x NC8889-2A	1800	1,33	1,67	1,33	0,33	1,00	0,00
S1	Arabella x T71	1800	4,67	1,33	0,67	0,00	0,00	0,00
S2	Tybalt x T71	1800	1,00	0,67	0,00	0,00	0,00	0,33
S3	Mandaryna x T71	1800	1,33	1,67	1,00	0,00	0,33	0,33
S4	Toridon x T71	1800	1,67	0,00	0,33	0,67	0,67	0,00
S5	T68xDC356/08-4-5/09	1800	8,00	4,67	7,33	0,00	0,33	1,00
S6	T71xDC356/08-4-5/09	1800	4,33	5,00	5,33	3,33	1,33	1,00

**Table 4:** Composition of growth hormones and various concentrations of ZEN in induction medium.

Component (mL/L)	Treatment					
	D0	D1	D2	K0	K1	K2
ZEN	-	0.1	0.2	-	0.1	0.2
2,4-D	1.0	1.0	1.0	1.5	1.5	1.5
Dicamba	1.0	1.0	1.0	-	-	-
Kinetin	-	-	-	0.5	0.5	0.5
